# The Heat-Induced Gel–Sol Transition in Coated Tofu: A Study on Protein Conformation and Microstructural Changes

**DOI:** 10.3390/gels11040237

**Published:** 2025-03-24

**Authors:** Xin Xie, Meng Li, Xinrui Diao, Saihua Sun, Ming Wen, Xiaohu Zhou, Liangzhong Zhao, Yang Li, Ping Lv, Bin Li, Xiaolong Shen, Zhanrui Huang, Hao Chen, Kuilin Zhang

**Affiliations:** 1 College of Food and Chemical Engineering, Shaoyang University, Shaoyang 422000, China; xiexin010106@163.com (X.X.); lm18987660976@163.com (M.L.); xinruid05@163.com (X.D.); 17353955124@163.com (S.S.); sys169@163.com (L.Z.); zhangrui_huang@163.com (Z.H.); spgc13@163.com (H.C.); zkl63@126.com (K.Z.); 2 Hunan Provincial Key Laboratory of Soybean Products Processing and Safety Control, Shaoyang 422000, China; 3 Hunan Engineering Research Center of Green Processing and Equipment of Hunan-Style Food, Shaoyang 422000, China; 4 College of Food Science, Northeast Agricultural University, Harbin 150030, China; liyanghuangyu@163.com (Y.L.); 13588028445@163.com (P.L.); libin.tx@163.com (B.L.); 15906837769@139.com (X.S.); 5 Zhejiang Daoji Agricultural Science and Technology Development Co., Ltd., Jiaxing 314000, China

**Keywords:** coated tofu, heat treatment, gel–sol

## Abstract

To enhance and stabilise the edible quality of coated tofu, this study explored the changes in the microstructure and intermolecular forces of coated tofu gel and sol under different heat treatments. It elucidated the phase transformation mechanism of coated tofu gel and sol under heat treatment. The results showed that the protein structure unfolded, the fluorescence intensity decreased, and the protein solubility, surface hydrophobicity, and free sulfhydryl group content increased as the coated tofu gel transformed to sol. Disulfide bonding and hydrophobic interactions were the primary intermolecular forces in the heat-induced gel–sol transition. FTIR showed that the content of β-sheets decreased significantly during gel–sol transformation, while the content of β-turns, α-helices and random coils increased significantly. Most remained relatively stable during the gel–sol transformation process, with only the A and B subunits of the 11S protein decreasing slightly. Their reduction became significant when the temperature reached 200 °C. Additionally, the high-temperature heat treatment promoted the gel–sol transition of the coated tofu, with its cross-section gradually transforming from a porous network structure to a more uniform and smooth texture during heat treatment process. The findings of this study provide a theoretical basis for improving the quality of coated tofu by optimising heat treatment parameters, laying the groundwork for future advancements in the development of pre-heat-treated coated tofu.

## 1. Introduction

With the evolution of contemporary lifestyles, consumer demands for enhanced quality and sensory characteristics in tofu products are intensifying, thereby stimulating interest in coated tofu [1]. Coated tofu, also known as “bomb tofu”, represents a regional delicacy in Southwest China. The thermal processing employed yields a distinctive golden, crisp exterior, contrasting a soft, creamy interior. This textural duality significantly enhances consumer appeal. The main aspects of traditional tofu processing include soybean soaking, grinding, filtration, adding coagulant, and moulding, etc., while coated tofu processing also includes acid discharge and alkali bubbling [2]. Coated tofu is essentially the product of soybean protein gelling [3], where the internal gel has transformed into a sol state, similar to tofu curd or soybean milk during heating. The taste of coated tofu is more delicate than that of traditional tofu.

A gel is a semi-solid substance composed of a three-dimensional network structure, exhibiting no fluidity, whereas a sol is a liquid colloidal system formed by a dispersed phase within a continuous phase, displaying a semi-fluid state [4]. Current research on protein gels is mainly focused on the sol–gel formation mechanism [5,6,7,8]. In the field of food, only preserved egg/salted egg (egg white protein/egg albumin) has been studied regarding the phase transition mechanism of gel to sol [9,10]; there are few reports on the gel–sol transition of soybean protein. Additionally, the gel–sol transformation of preserved egg/salted egg negatively impacts its quality—it causes deterioration [11]—while the gel–sol transformation of coated tofu results in a more layered tofu flavour—a kind of optimisation. Therefore, the molecular mechanism of the gel–sol transformation of coated tofu is in urgent need of systematic study.

A gel is a semi-solid substance composed of a three-dimensional network structure, exhibiting no fluidity, whereas a sol is a liquid colloidal system formed by a dispersed phase within a continuous phase, displaying a semi-fluid state [12]. Protein gel–sol transition is a dynamic process that is susceptible to external environmental interference from temperature [12], pH [13], ionic strength [14], additives, etc., which affect the degree of gel network dispersion. Previous studies have revealed that heat treatment is a critical factor influencing the texture of coated tofu. Various heat treatment methods, such as microwaving, baking, and air frying, can induce a gel–sol transition in coated tofu. Among these methods, air-fried coated tofu exhibits superior overall quality compared to deep-frying and baking. Heat treatment significantly affects the extent of the gel–sol transition. Insufficient temperature or short processing time may prevent the tofu gel from transitioning into a sol, whereas excessive temperature or prolonged processing can lead to a reduction in the internal sol content, resulting in energy waste and contradicting the principles of sustainable development. At present, the research on coated tofu is limited to the identification of flavour substances and spoilage bacteria [1]; there have been no relevant studies on the effect of heat treatment on the alkali-induced phase transition mechanism of coated tofu.

Accordingly, this study explored the mechanism of protein molecular conformational transformation during the gel–sol formation process of coated tofu, as well as the process of protein gel–sol degradation. The protein components, intermolecular forces, surface properties, and microstructure of coated tofu were investigated under varying heat treatment durations and temperatures. This study elucidated the effects of heat treatment on the gel–sol phase transformation mechanism of this product. The findings address a research gap in the field and provide a theoretical foundation for enhancing the quality stability of coated tofu. These insights also support the development of a new industry focused on pre-heat-treated coated tofu.

## 2. Results and Discussion

### 2.1. Protein Solubility

**Figure 1:**
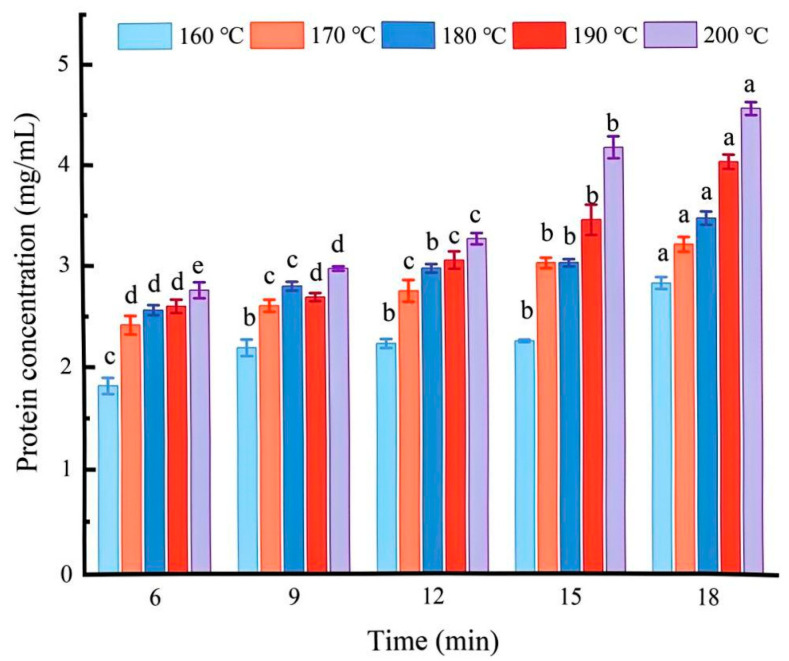
Changes in soluble protein content of coated tofu during heat treatment. Different letters at the same temperature indicate significant differences (*p* < 0.05).

The solubility of protein can reflect the degree of protein degradation and aggregation and can influence its gelation properties [15]. As Figure 1 shows, during the heat treatment of coated tofu, the soluble protein concentration increased as the temperature increased. At 160 °C, the solubility of coated tofu increased from 1.82 ± 0.08 to 2.83 ± 0.05 mg/mL, and at 200 °C, the solubility increased from 2.77 ± 0.07 to 4.56 ± 0.06 mg/mL. This may have been due to the dissociation of protein aggregates during the heat treatment process. This destroyed the gel structure of the coated tofu and transformed it into a sol-like structure with increased fluidity in the high-temperature alkaline environment, increasing the protein solubility [16].

### 2.2. The Protein Composition Was Analysed Using Sodium Dodecyl Sulphate-Polyacrylamide Gel Electrophoresis (SDS-PAGE)

**Figure 2:**
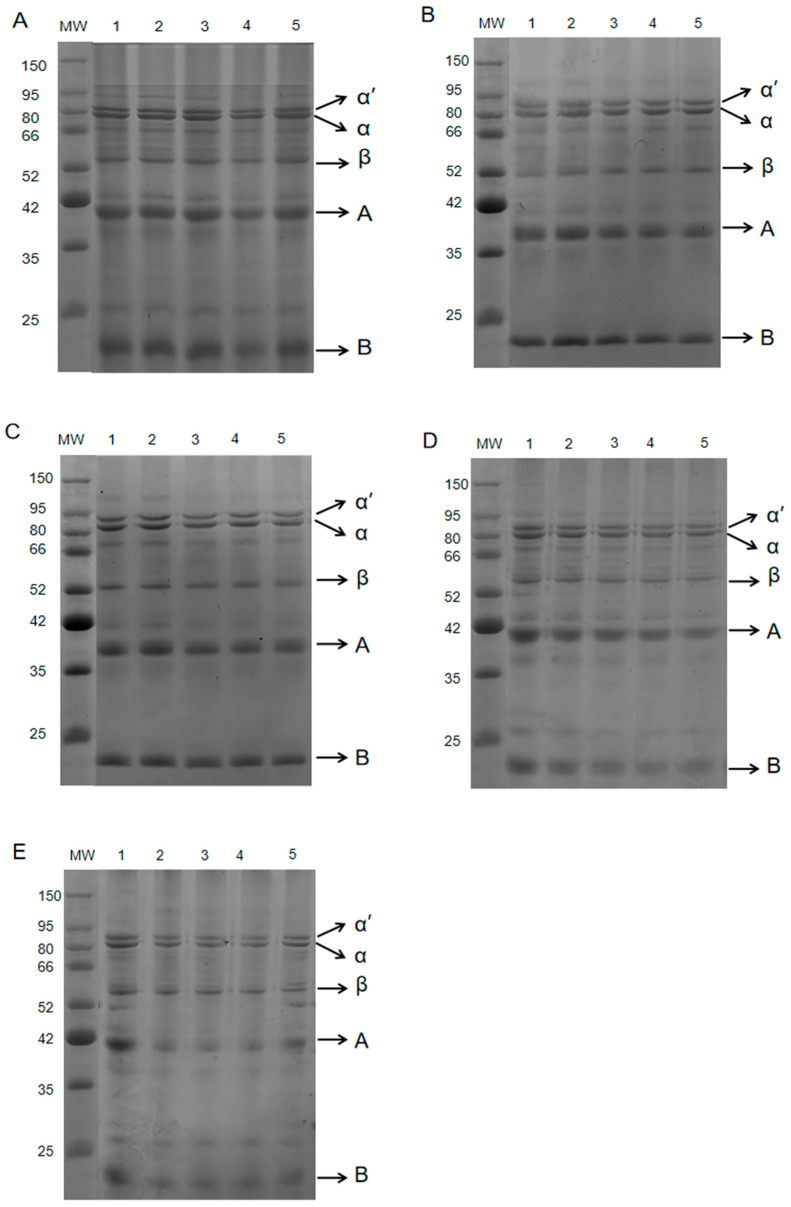
SDS-PAGE results showing the effect of heat treatment on coated tofu protein subunits. Note: (**A**–**E**) correspond to heat treatment at 160, 170, 180, 190, and 200 °C, respectively; 1–5 correspond to heat treatment durations of 6, 9, 12, 15, and 18 min, respectively.

SDS-PAGE can separate proteins according to the different molecular weights of protein subunits, so it is often used to detect changes in protein components [17]. Figure 2 shows that the protein molecular weight range of the coated tofu was 20–71 kDa. The two electrophoretic bands at 66–80 kDa corresponded to the α’ and α subunits of the 7S protein, and the bands near 50 kDa corresponded to the β subunits of the 7S protein. The 38 and 20 kDa bands corresponded to two low molecular weight subunits, acid polypeptide A and basic polypeptide B, which formed by the cleavage of the 11S protein disulfide bond [18]. As Table 1 shows, during the 160–180 °C heat treatments most of the subunits were relatively stable, with only the A and B subunits of the 11S protein decreasing slightly. At 190 °C, the A and B subunit bands of 11S globulin gradually became fainter, and as the heating time increased from 6 min to 18 min, the A and B subunits decreased by 2.63% and 5.69%, respectively. At 200 °C, the A and B subunit content decreased significantly, with, respectively, reductions of 9.66% and 8.50%. This phenomenon may be attributed to the polymerisation of 11S globulin at very high temperatures, forming soluble aggregates that cannot enter the gel [19]. Li et al. [7] also found that an increase in heating intensity resulted in a gradual decrease or the complete loss of the A and B subunits.

### 2.3. Surface Hydrophobicity

**Figure 3:**
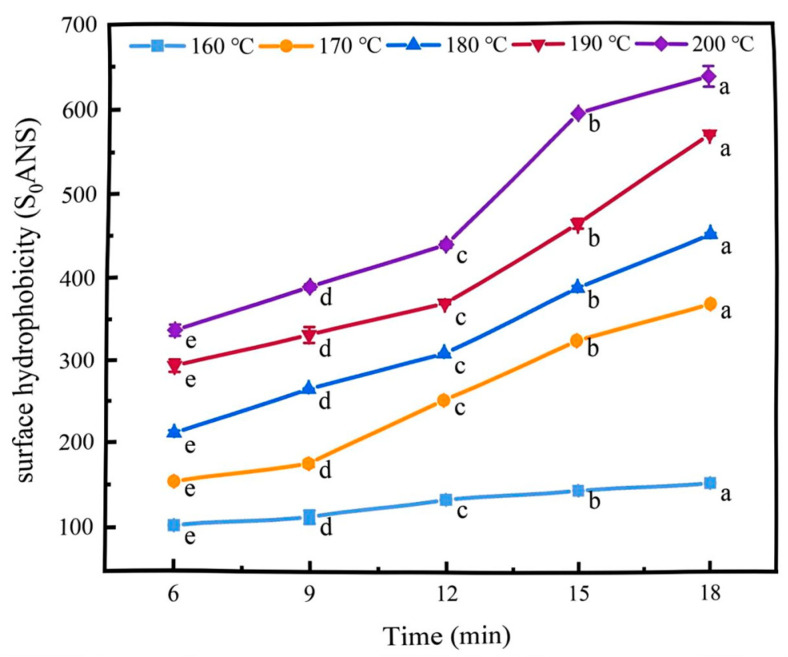
Effect of heat treatment on surface hydrophobicity of coated tofu, Different letters at the same temperature indicate significant differences (*p* < 0.05).

Hydrophobicity refers to the tendency of non-polar molecules to associate with each other in a polar environment [20]. The surface hydrophobicity index can characterise the relative content of hydrophobic groups on the surface of protein molecules and is an important parameter for evaluating protein conformational changes [21]. As shown in Figure 3, the surface hydrophobicity of the coated tofu increased as the temperature increased, rising from 157 ± 1.59 to 634.47 ± 12.6 after 18 min of heat treatment. This may have been because the cross-linked structure of the coated tofu was gradually destroyed as the temperature increased, exposing the internal hydrophobic active groups and increasing its surface hydrophobicity [22]. Simultaneously, as the heating duration increased, the surface hydrophobicity of the coated tofu also increased (*p* < 0.05). For instance, at 160 °C, the surface hydrophobicity increased by 56.17 when the heating time was extended from 6 min to 18 min, whereas at 200 °C, it increased by 299.57. This may have been because the gel was severely liquefied so the gel network structure completely collapsed and the hydrophobic groups buried in the protein molecules were exposed [23].

### 2.4. Fourier Transform Infrared (FTIR) Spectroscopy Was Used to Analyse Changes in Secondary Structure

**Figure 4:**
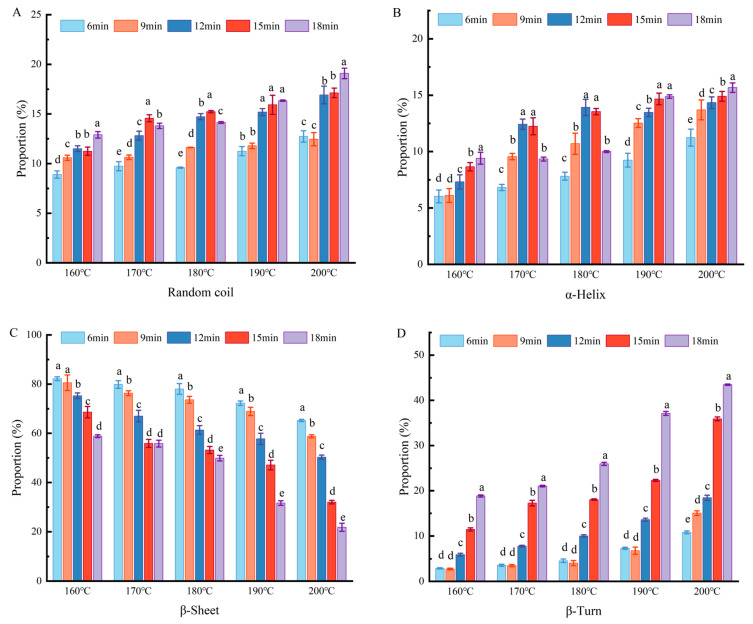
Influence of heat treatment on coated tofu secondary structure. Note: (**A**–**D**) represent random coils, α-helixes, β-sheets, and β-turns, respectively. Different letters at the same temperature indicate significant differences (*p* < 0.05).

Infrared spectroscopy can be used to identify changes in protein structures [24]. The secondary structure of tofu changed markedly under different heat treatments. As shown in Figure 4, when the temperature increased, the β-sheet content decreased significantly, while the content of β-turns, α-helices, and random coils increased significantly. After 18 min of heat treatment, the β-sheet content decreased from 58.84% at 160 °C to 21.78% at 200 °C, whereas the α-helix content increased from 9.41% at 160 °C to 15.67% at 200 °C. This indicates that the ordered structure began to transition into a disordered state. However, the content β-sheet and β-turn (β-sheets + β-turns > 60%) always remained consistently higher than that of α-helices and random coils, indicating that the sol structure was mainly composed of ordered proteins. These findings are consistent with the trends observed by Fan et al. in their study on the hydration mechanism of duck egg gels under high temperatures [22]. There was a gradual increase in α-helical structures, further confirming that heat-induced protein structural unfolding occurred [25]. The increase in random curl content indicated that the structure became looser and more disordered, improving the solubility of the coated tofu [26]; this was consistent with the results of protein solubility. These secondary structural changes also indicated that the gel–sol transformation of coated tofu was related to structural changes in proteins, with the gel–sol process accompanied by the disintegration of β-sheet structure aggregates. Some studies have shown that the β-sheet structure is particularly important for gel stability, with more β-sheets promoting greater aggregation [27].

### 2.5. Fluorescence Spectroscopy Was Employed to Analyse Changes in the Tertiary Structure of Proteins

The endogenous fluorescence of proteins is caused by the aromatic amino acids tryptophan, tyrosine, and phenylalanine. The fluorescence emission of tryptophan is often used as an indicator of conformational changes in proteins because it is susceptible to the local environment. Therefore, the fluorescence intensity and maximum emission wavelength (λmax) in the endogenous fluorescence spectra of proteins can reflect the aqueous environment around tryptophan residues, providing important information about the tertiary protein structural conformation [28].

**Figure 5:**
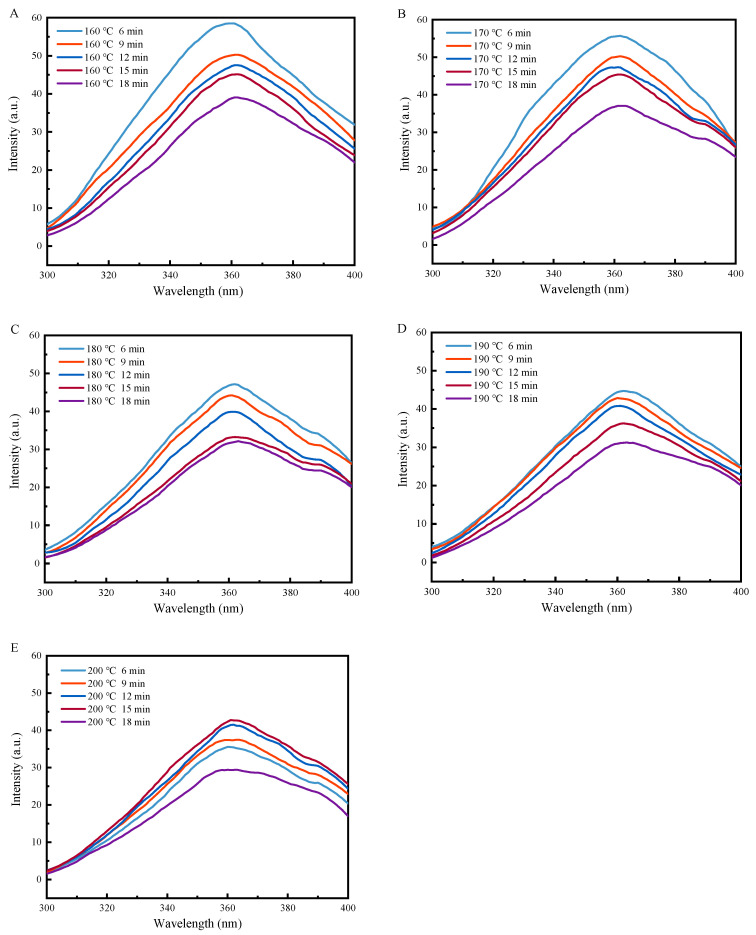
Effect of heat treatment on fluorescence of coated tofu. Note: (**A**–**E**) correspond to coated tofu after heat treatment at 160, 170, 180, 190, and 200 °C, respectively.

As shown in Figure 5, as the heat treatment duration increased, the fluorescence intensity of the coated tofu gradually decreased. The decrease in fluorescence intensity was accompanied by a long-wave shift in the λmax position, showing a redshift phenomenon. For instance, at 160 °C, the λmax shifted from 359 nm to 363 nm, indicating an enhancement of the polarity of the aqueous environment where the tryptophan group was located, similar to that observed in heat-treated soybean protein [29]. This phenomenon was attributed to the further development of the protein structure caused by heat treatment, exposing the tryptophan residues buried inside the protein to a polar aqueous environment [30].

Additionally, as the heat treatment temperature increased, the fluorescence intensity gradually decreased. The fluorescence quenching efficiency decreased from 33.21% at 160 °C to 16.28% at 200 °C after 6 min of heat treatment, which indicated that the tryptophan residues of the protein molecules were less exposed during higher temperature treatments. The results of surface hydrophobicity measurements also showed that an increase in the heat treatment temperature accelerated protein molecule dispersion. Therefore, it was speculated that the decrease in fluorescence intensity may have been due to the protein molecule peptide chains preferentially surrounding peptide chains containing tryptophan residues, where they formed aggregates through hydrophobic- and disulfide-bonds at high temperatures. As a result, the fluorescence intensity was lower than for the lower-temperature treated samples [31].

### 2.6. Intermolecular Forces

**Figure 6:**
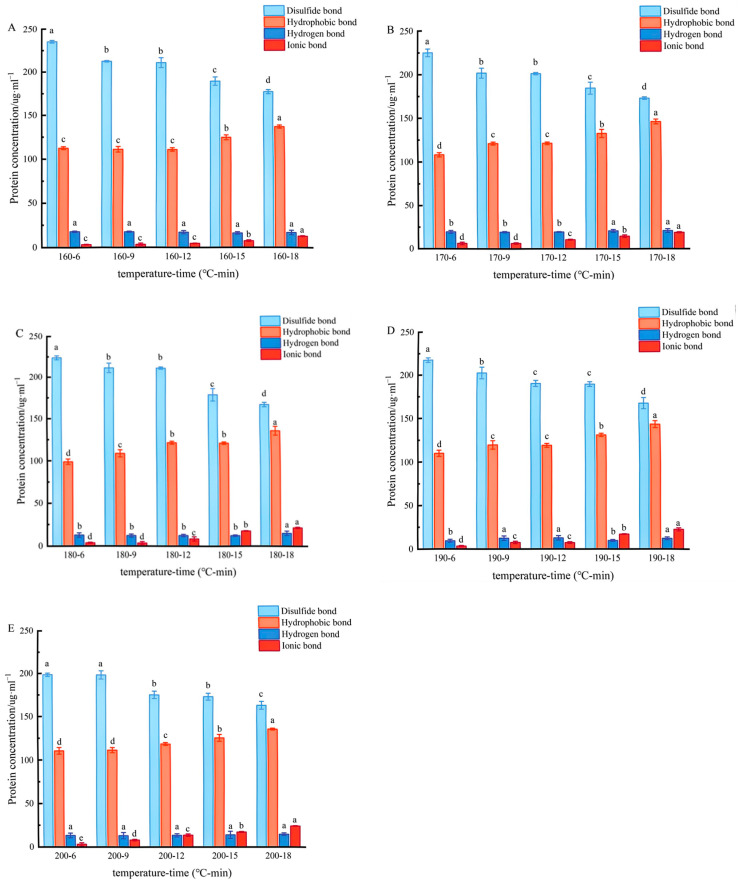
Effect of heat treatment on the intermolecular forces of coated tofu. Note: (**A**–**E**) correspond to coated tofu after heat treatment at 160, 170, 180, 190, and 200 °C, respectively, Different letters at the same temperature indicate significant differences (*p* < 0.05).

The high-level structure of a protein is subjected to pyrolysis folding, which leads to aggregation, crosslinking, and the re-establishment of intermolecular forces. Specific chemical agents can destroy these forces, so the contribution of intermolecular forces in protein gels can be characterised by determining protein solubility in different chemical agents [32]. As shown in Figure 6, the relative contribution of the four main intermolecular forces relevant to coated tofu was as follows: disulfide bonds > hydrophobic bonds > hydrogen bonds > ionic bond. When heated at 160 °C for 6 min, the concentrations of disulfide bonds and hydrophobic interactions were 235 mg/mL and 112 mg/mL, respectively, while the concentrations of hydrogen bonds and ionic bonds were only 17 mg/mL and 3 mg/mL, respectively. This indicates that disulfide bonds and hydrophobic interactions predominated in the heat-induced gel–sol transition process.

In the present study, in general, the effect of the heat treatment temperature on intermolecular forces was relatively limited and the effect of the heat treatment duration was relatively pronounced. As the heating duration increased, the original alkali-denaturised coated tofu underwent further thermal denaturation, disintegrating cross-linked proteins, destroying the gel system balance and intensifying negative ionisation on the surface of protein molecules, resulting in an increase in ionic bonding [33]. Disulfide bonds, important components of the system that often contribute to gel formation and stability, are easily broken when in a high pH for a long time [33]. Therefore, continuous heating resulted in a progressive reduction in the proportion of disulfide bonds under the continuous action of strong alkali because it aggravated the infiltration of alkali into the coated tofu [34]. It has previously been reported that the breaking of disulfide bonds can change the conformation of protein molecules, reduce the average particle size [35], and loosen the structure. Thus, all of these effects promoted the conversion of gel to sol.

### 2.7. Free Sulfhydryl Groups

Protein sulfhydryl groups are the most active groups in protein. Disulphide covalent bonds form after oxidation, which are strong protein cross-linking forces [36]. In general, the more free sulfhydryl groups that are exposed, the stronger the intermolecular interactions and the stronger the gel properties of a protein [37].

**Figure 7:**
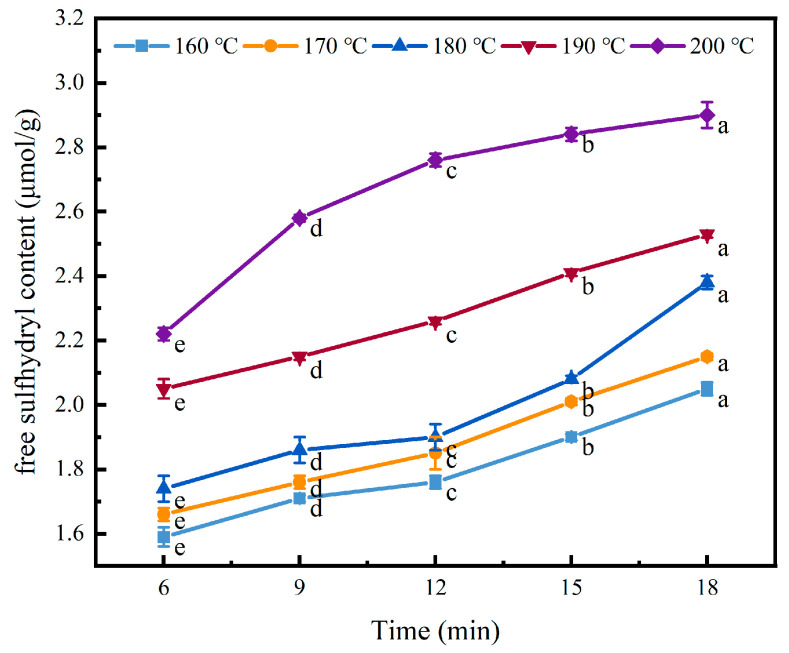
Effect of heat treatment on the free sulfhydryl content of the coated tofu. Different letters at the same temperature indicate significant differences (*p* < 0.05).

As shown in Figure 7, with the increase in heat treatment temperature, the free sulfhydryl (SH) content exhibited a significant upward trend (*p* < 0.05). After 18 min of heat treatment, the free SH content increased from 2.05 μmol/g at 160 °C to 2.9 μmol/g at 200 °C. At 160–180 °C, the content of free sulfhydryl groups increased slowly when the heating duration was less than 12 min, after which the content increased significantly. At 190–200 °C, the increase in free sulfhydryl groups was more significant. This indicates that considerable chain disintegration and disulphide bond disruption occurred under heat treatment, exposing the free sulfhydryl groups in the original molecule and thereby increasing the content of free sulfhydryl group. As the heat treatment time was extended, the content of free sulfhydryl groups also increased. This is due to the expansion of the internal structure of the protein, the dissociation of protein subunits, and the breaking of disulfide bonds in protein molecules [38]. Heat treatment tends to promote the oxidation of free sulfhydryl (SH) groups, leading to the formation of disulfide bonds. However, under high temperatures (>90 °C) and alkaline conditions, the sulfhydryl–disulfide exchange reaction readily occurs, shifting the equilibrium toward the generation of free sulfhydryl groups [39].

### 2.8. Scanning Electron Microscopy (SEM) Was Used to Analyse Microstructural Changes

**Figure 8:**
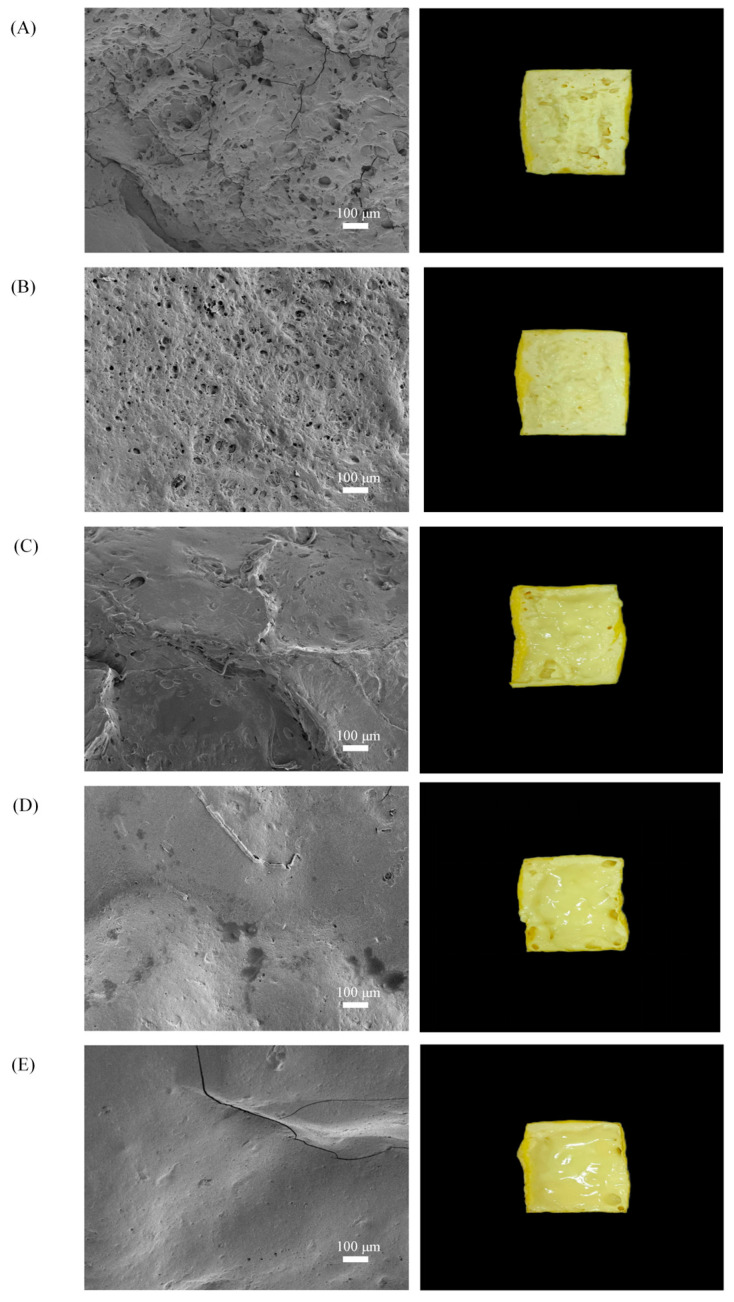
Effect of heat treatment on the microstructure of coated tofu. Note: (**A**–**E**) represent coated tofu after heating at 180 °C for 6, 9, 12, 15, and 18 min, respectively. The right side shows the internal state of coated tofu after heat treatment at 180 °C, with a magnification of 100×.

SEM is an important technique for studying microstructures such as three-dimensional network structures, aggregates, and other spatial arrangements [40]. As shown in Figure 8, after the heating time was extended from 6 to 9 min, the coated tofu had a more porous network structure due to increased water loss, which resulted in the formation of more honeycomb-like holes [41]. When the heating time was extended from 9 to 18 min, the microscopic pores of the coated tofu cross-section gradually became smaller, smoother, and more uniform. This may have been due to a change in intermolecular forces under strong alkali action. During the heating process, the cross-linked protein in the coated tofu gel disintegrated, the gel structure became looser [42], and the active groups were exposed, forming the sol. After cooling, the sol reaggregated to form a uniform and stable three-dimensional network structure, so its cross-section was relatively smooth. Thus, the thermal modification improved the formation of a uniform network.

## 3. Conclusions

In this study, the effect of heat treatment on the phase transition mechanism of coated tofu gel and sol was investigated by observing changes in the microstructure and intermolecular forces of the gel and sol. The results demonstrate that the gel–sol transition in coated tofu is closely associated with heat-induced protein unfolding, as evidenced by the increase in protein solubility and α-helix content. During the gel–sol transition, high heat treatment intensity enhanced the infiltration of lye, leading to alterations in secondary structures. The structure of coated tofu became looser and more fluorescent groups were exposed on the surface. This resulted in a reduction in fluorescence intensity, an increase in protein solubility and surface hydrophobicity, and the disruption of intermolecular forces, including disulphide bonds and hydrogen bonds. Furthermore, SDS-PAGE results indicate that the transition is accompanied by changes in subunits, with the bands of the A and B subunits of the 11S globulin gradually fading over time. SEM results reveal that the porous network structure becomes more uniform and smoother during the gel–sol transition. This study focused solely on air-frying as a heat treatment method. Future research could investigate different heat treatments (e.g., deep-frying, baking), varying pH conditions, or different ionic environments to explore protein cross-linking behaviours, thereby providing deeper insights into the gel–sol transition mechanism of coated tofu.

## 4. Materials and Methods

### 4.1. Materials

The soybeans used were commercialised Anhui soybeans (Anhui, China). The following reagents were purchased: sodium chloride, methanol, and glacial acetic acid from Tianjin Yongda Chemical Reagent Co., Ltd. (Tianjin, China); sodium hydroxide from Xilong Science Co., Ltd. (Shantou, China); disodium hydrogen phosphate, from Jiangyin Xingxin Biotechnology Co., Ltd. (Wuxi, China); potassium dihydrogen phosphate, from Henan Jinchuan Fertiliser Co., Ltd. (Xinyang, China); potassium chloride and potassium bromide, from Sinopsin Group Chemical Reagent Co., Ltd. (Beijing, China); urea, from Xilong Science Co., Ltd. (Shantou, China); β-mercaptoethanol, from Sinopsin Group Chemical Reagent Co., Ltd. (Beijing, China); potassium bromide and sodium dodecyl sulphate (SDS), from Tianjin Kemiou Chemical Reagent Co., Ltd. (Tianjin, China); BCA protein assay kit and Bradford protein assay kit, from Isejo Biotechnology Co., Ltd. (Jiangsu, China); and electrophoresis reagents from Shanghai Biyuntian Biotechnology Co., Ltd. (Shanghai, China). All reagents were analytically pure.

### 4.2. Preparation of Coated Tofu

After cleaning and removing impurities, soybeans were soaked for 8 to 12 h. The soaked soybeans were ground with water at a bean-to-water ratio of 1:8, and the resulting slurry was filtered to obtain cooked soymilk. The soymilk was heated to 85 °C, and coagulants (0.3% gypsum, 0.3% MgCl_2_, and 25% soybean whey fermentation broth, all percentages based on the weight of the soymilk) were added. The mixture was allowed to settle for 10–15 min, after which the curd was broken and poured into moulds for pressing and shaping. The formed tofu was cut into pieces and soaked in an alkaline solution (2% NaHCO_3_, 0.8%NaCl, pH 8.0, the dosage is calculated based on the weight of water) to produce coated tofu.

### 4.3. Heat Treatment of Coated Tofu

The air fryer was preheated for 5 min. Then, 15 g of uniformly coated tofu (3 × 4 cm) was place into the air fryer and it was ensured that each piece was centrally positioned. The coated tofu pieces were evenly positioned in the centre of the fryer. The tofu was heated in the air fryer at 160, 170, 180, 190, or 200 °C for 6, 9, 12, 15, or 18 min, and some samples were subjected to freeze-drying.

### 4.4. Determination of Soluble Protein Content

The method of Xue et al. [43] was followed with minor modifications. After heat treatment, the outer shell of the coated tofu was removed, and 3 g of coated tofu gel–sol from the central portion was added to 27 mL of PBS buffer (0.01 mol/L, pH 8.0), homogenised at 10,000 rpm for 2 min and then centrifuged at 8000 rpm for 20 min. Using bovine serum albumin (BSA) as the standard, the protein content of the supernatant was measured at 562 nm using the BCA assay.

### 4.5. Sodium Dodecyl Sulphate Polyacrylamide Gel Electrophoresis (SDS-PAGE)

The method of Kudre et al. [44] was followed with minor modifications. After heat treatment, the outer shell of the coated tofu was removed, and 1 g of coated tofu gel–sol from the central portion was weighed, mashed, and mixed with 9 mL of 5% (*w/v*) SDS solution. The mixture was heated in an 85 °C water bath for 1 h. The solution was then cooled and centrifuged at 8000 rpm for 10 min, and the supernatant was removed and diluted to a 2 mg/mL protein concentration. The diluted supernatant was mixed with protein sampling buffer (5×), boiled in a water bath for 5 min, and then cooled and centrifuged at 10,000 rpm for 5 min. A 10 μL aliquot was taken for sampling.

For electrophoresis, 5% concentrated glue and 12% separation glue were used, with voltages at 80 V and 120 V, respectively. The gel was dyed with Coomassie bright blue for 45 min and then eluted with the decolorizing solution.

### 4.6. Surface Hydrophobicity Measurement

The method of Xin [45] was followed with modifications. After heat treatment, the outer shell of the coated tofu was removed, and the coated tofu gel–sol from the central portion was freeze-dried. The freeze-dried sample (0.1 g) sample was dissolved in PBS (0.01 mol/L, pH 8.0, 10 mL), magnetically stirred at 25 °C for 1 h, and then centrifuged at 8000 rpm for 10 min. The protein concentration in the supernatant was measured by the Bradford method, with the supernatant diluted by gradient to ensure that the concentration was within the range of 0.005–0.5 mg/mL. To the protein solution (4 mL), 40 μL ANS was added. After thorough mixing, the sample was allowed to stand for 3 min, and its fluorescence intensity was measured using a G9800A fluorescence spectrophotometer (Cary Eclipse, Agilent, Santa Clara, CA, USA). The excitation wavelength (λex) was 370 nm, the emission wavelength (λex) was 490 nm, and the slit width was 5 nm. With protein mass concentration as the horizontal coordinate and sample fluorescence intensity as the vertical coordinate for linear fitting, the slope in the initial stage was the surface hydrophobicity index.

### 4.7. Fourier Transform Infrared Spectroscopy

The method of Xu et al. [46] was followed with a slight modification. After heat treatment, the outer shell of the coated tofu was removed, and the coated tofu gel–sol from the central portion was freeze-dried. An appropriate amount of the freeze-dried sample was uniformly mixed with KBr at a ratio of 1:50 and then pressed into a transparent sheet with a thickness of 1 mm. Each sheet was analysed by Fourier transform infrared spectrometry (Nicolet iS5, Thermo Fisher Scientific, Shanghai, China) in the range of 4000–500 cm^−1^, with 64 scans and a resolution of 4.0 cm^−1^. The detection environment required that the operating temperature be controlled at 25 °C and the relative humidity at 50%. Baseline correction was performed using OMNIC 9.7 software, and the data were saved in CSV format. Using PeakFit v4.12, the peak area of the amide I band (1600–1700 cm^−1^) was used to indicate the relative content of the secondary protein structure.

### 4.8. Fluorescence Spectrometry

This was performed according to the method of Wang et al. [47], with a slight modification. After heat treatment, the outer shell of the coated tofu was removed and 1 g of the coated tofu gel–sol from the central portion was weighed and mixed with 9 mL of PBS buffer (0.01 mol/L, pH 8.0). The mixture was homogenised at 12,000 rpm for 2 min and then centrifuged at 10,000 rpm for 15 min at 4 °C. The supernatant was collected and diluted to a concentration of 0.2 mg/mL. The fluorescence intensity of the sample was then measured using a G9800A fluorescence spectrophotometer (Cary Eclipse, Agilent, Santa Clara, CA, USA). The excitation wavelength was 290 nm, the emission wavelength was 300–400 nm, and the slit width was 5 nm. All the measurements were repeated three times.

### 4.9. Determination of Intermolecular Force

This was performed based on the method of Xue et al. [48] with a slight modification. After heat treatment, the outer shell of the coated tofu was removed, and five 1 g portions of the coated tofu gel–sol from the central portion were weighed. They were then mixed with 10 mL of 0.05 mol/L NaCl (SA), 0.6 mol/L NaCl (SB), 0.6 mol/L NaCl + 1.5 mol/L urea (SC), 0.6 mol/L NaCl + 8 mol/L urea (SD), or 0.6 mol/L NaCl + 8 mol/L urea + 0.2 mol/L 2-mercaptoethanol (SE). The solution was mixed, then water was added to 50 mL and centrifuge at 10,000 rpm for 10 min. The supernatant was removed, and the protein content was determined using a Bradford kit. The difference between the protein content of SB and SA corresponded to the number of ionic bonds in the gel structure. The difference between the measured protein content of SC and SB corresponded to the number of hydrogen bonds. The difference between the protein content of SD and SC corresponded to the number of hydrophobic bonds. The difference between the protein content of SE and SD corresponded to the disulfide bond content.

### 4.10. Determination of Free Sulphhydryl Groups

This was performed referring to the method of Hu et al. [49], with a slight modification. After heat treatment, the outer shell of the coated tofu was removed, and the coated tofu gel–sol from the central portion was freeze-dried. The freeze-dried sample (0.1 g) was dissolved in 10 mL of Tris-glycine buffer (10.4 g Tris, 6.9 g glycine, and 1.2 g EDTA per litre, pH 8.0) at 4 °C. The supernatant was collected by centrifugation at 12,000× *g* for 15 min. Then, the supernatant (4 mL) and Ellman reagent (4 mg/mL DTNB prepared from Tris-glycine buffer, 40 μL) were mixed well, stored in the dark for 15 min, and the absorbance of the solution was determined at 412 nm. The free sulphydryl groups were calculated as follows:

SH (μM) = 73.53 × A412 × D/C

where 73.53 is 106/(1.36 × 104), A412 is the determined absorption value, D is the dilution ratio, and C is the protein concentration of the sample supernatant.

### 4.11. Scanning Electron Microscopy

This was performed referring to the method of Kang et al. [50]. After heat treatment, the outer shell of the coated tofu was removed and allowed to cool. A 3 mm cube was cut from the central portion and fixed with a 2.5% glutaraldehyde solution for 2 h. After fixation, the sample was rinsed 5–10 times with PBS buffer (0.1 mol/L, pH 7.2). Then, gradient dehydration was performed using 30%, 50%, 70%, 90%, and 100% ethanol for 10 min each and volatile organic solvents were removed with cold air in the fume hood. Each sample was freeze-dried for 15 h and then sputter-coated with gold for 45 s using an Oxford Quorum sputter coater (SC7620, UK) at a current of 10 mA prior to observation. The sample morphology was captured using a scanning electron microscope (TESCAN MIRA LMS, Czech Republic, Shanghai, China). For morphological imaging the acceleration voltage was set to 3 kV, while for energy-dispersive spectroscopy (EDS) mapping the acceleration voltage was adjusted to 15 kV.

### 4.12. Statistics and Analysis

Data processing was performed using Origin 2018, and statistical analysis was conducted using SPSS 25.0. One-way analysis of variance (ANOVA) followed by Duncan’s multiple range test (*p* < 0.05) was employed to assess statistical differences between groups. The results are expressed as the mean ± standard deviation (SD). All data were measured in triplicate.

## Figures and Tables

**Table 1:** The band intensity of reduced proteins in coated tofu under different heat treatments.

Temperature-Time (°C-min)	α’	α	β	A	B
160-6	10.20%	11.69%	9.26%	17.89%	20.90%
160-9	10.34%	11.58%	9.20%	17.66%	20.41%
160-12	10.18%	11.72%	9.30%	17.61%	20.18%
160-15	10.20%	11.50%	9.29%	17.21%	20.20%
160-18	10.11%	11.79%	9.47%	17.08%	19.54%
170-6	10.10%	11.65%	9.30%	17.50%	20.35%
170-9	10.83%	11.72%	9.27%	17.44%	20.65%
170-12	10.70%	11.44%	9.44%	17.25%	19.80%
170-15	10.20%	11.76%	9.59%	17.09%	19.29%
170-18	10.45%	11.20%	9.83%	16.96%	19.20%
180-6	10.41%	11.22%	9.63%	17.49%	19.96%
180-9	10.65%	11.45%	9.86%	17.41%	19.61%
180-12	10.15%	11.81%	9.89%	17.17%	19.75%
180-15	10.87%	11.62%	9.79%	17.12%	19.16%
180-18	10.83%	11.58%	9.61%	16.92%	18.87%
190-6	10.79%	11.01%	9.43%	17.16%	18.74%
190-9	10.57%	11.85%	9.26%	16.57%	16.20%
190-12	10.76%	11.75%	9.30%	15.77%	15.17%
190-15	10.60%	11.74%	9.32%	15.06%	13.99%
190-18	10.70%	11.75%	9.19%	14.53%	13.05%
200-6	10.65%	11.49%	9.00%	17.80%	17.93%
200-9	10.70%	11.46%	8.68%	14.97%	13.33%
200-12	10.56%	11.50%	8.91%	9.67%	11.31%
200-15	10.68%	11.52%	8.75%	7.89%	9.02%
200-18	10.59%	11.53%	8.89%	8.14%	9.43%

## Data Availability

The original contributions presented in this study are included in the article. Further inquiries can be directed to the corresponding authors.
